# High-Throughput Sequencing Detects a Viral Complex in *Agave Tequilana* Plants

**DOI:** 10.1155/av/6434701

**Published:** 2025-05-04

**Authors:** Rodolfo De La Torre-Almaraz, Héctor Salgado-Ortiz, Mario Salazar-Segura

**Affiliations:** Laboratorio de Fitopatología, UBIPRO FES IZTACALA. Unam, Av. de Los Barrios 1, Hab. Los Reyes Ixtacala, Tlalnepantla de Baz 54090, Mexico

**Keywords:** Mexico, RNA virus, tequila, tymovirales, vitivirus

## Abstract

*Agave tequilana* Weber var. Azul is one of the most economically important species in Mexico because of its use in the production of tequila. Recently, young agave plants in commercial plantations in the state of Jalisco and agave ornamental plants have been observed to have symptoms of yellow streaks and mottle like those caused by viruses. The diversity of symptoms observed in agave and the negative results of the different diagnostic tests indicated the possible presence of different unknown viruses, and so we conducted high-throughput sequencing (HTS) analyses of viral RNA. The bioinformatics analyses showed the complete genomes of isolates of *Vitivirus* genus, *Potexvirus* genus, *Tepovirus* genus, and the partial genome of a *Badnavirus* genus in mixed infections in agave samples from commercial plots and in ornamental plants. The presence of each virus was confirmed by sequencing and cloning of the RT-PCR products of the capsid protein (CP), using specific oligonucleotides designed from the sequences obtained by HTS. This is the first time to our knowledge that mixed infections of potential novel *Vitivirus, Potexvirus, Tepovirus*, and *Badnavirus* genomes have been identified in *A. tequilana* plants.

## 1. Introduction

*Agave* (Family: Asparagaceae) is a genus of monocotyledon, succulent plants native to the arid regions of central and northern Mexico and southern United States. It includes more than 200 species, around 150 of which are distributed in Mexico, which is therefore considered the center of origin and primary diversification of the *Agave* genus. Some references identify more than 518 species of native agaves distributed in Mexican territory [1] and estimate that this genus began its diversification some 12 million years ago. Therefore, Mexico is considered the center of origin and primary diversification of the *Agave* genus [2]. Depending on the region, the different species of agave are known by their local names, such as agave, pita, maguey, cabuya, fique, or mezcal, among the most common. Since pre-Hispanic times, it has been used as a source of fiber to make clothes and blankets and as roofing; some are used to produce syrups, candies, and other foods, while others are used as ornamental plants in public and private gardens. The most widely known use is in the production of alcoholic beverages: pulque as well as distilled mezcal and tequila [3, 4]. *Agave tequilana* Weber var Azul is one of the most economically important plants in Mexico because it is the raw material for making tequila, the alcoholic beverage with the greatest demand worldwide. In 2023, Mexico produced 472.2 million liters of tequila (100% Agave) and exported 420 million valued at 64 billion dollars [4, 5]. During field work in 2017 in commercial agave plantations and nurseries in the state of Jalisco and in Chapultepec National Park, Mexico City, we observed plants with symptoms consisting of thin light yellow streaks, which formed long broad spots when they fused, and mottle or irregular whitish-yellow spots that contrast with the blue-green ash color typical of this agave species, what we describe as “Agave yellow streak and mottle.” The spots eventually cause deformation and necrosis on the leaves. However, the damage disappears on new stems of adult plants, but in old leaves hard-to-see faint fine yellow streaks or mottle persist. The causal agent of those symptoms has not been previously described in plants grown under natural conditions in Mexico and are associated with damage caused by virus. The available bibliographic information in Mexico only describes pests and diseases caused by fungi or bacteria that affect the blue agave and generally indicates a disease such as yellow mottle as a possible viral disease, but they do not include additional biological confirmation on the identity of the supposed virus associated with the symptoms described [4, 6, 7]. A review of the sequences available in the GenBank (NCBI) revealed three sequences of nucleotides (DQ525858; DQ5255856; DQ525857), belonging to part of a region of the capsid protein (CP) gene (5949–6958 nt) of an RNA virus of the Order *Tymovirales* from agave samples. However, there was no additional information on the biological or morphological characteristics of the possible virus. Analysis of the available information did not allow us to determine the identity of possible viruses associated with agave in Mexico, and due to the diversity of the symptoms of streaks, mottle and irregular spots observed on the blue agave, a hypothesis was proposed that that these symptoms could be caused by unknown viruses. Tests for mechanical transmission to the host range and serological assays (Agdia ELISA Test kits for Potyvirus, TMV, CVX, and PVX, Elkhart, IN, USA) for were negative in all experiments (data not shown). However, observations of ultra sections of agave tissues with symptoms under a transmission electron microscope showed flexible rod-shaped viral particles [8].

The use of appropriate methods for diagnosing viral diseases is important to keep crops healthy and productive, as well as to avoid spreading these pathogens to other regions and other susceptible hosts and to implement timely phytosanitary measures. Considering its sensitivity [9–12], we used high-throughput sequencing (HTS) technology to identify the probable viruses associated with agave yellow stripe. This technique sequences millions of nucleotides in a short time, enabling detection of most viral pathogens in a sample.

## 2. Methodology

### 2.1. Collection of Material

**Figure 1:**
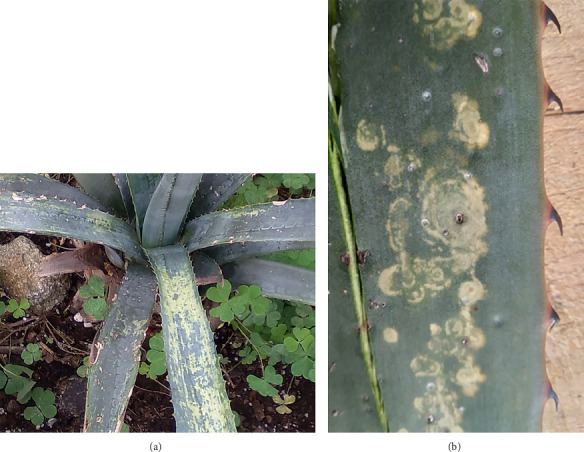
Yellow streak and mottle symptoms of viral origin in young blue agave plants. (a) Commercial plantation and nurseries, in the state of Jalisco, and (b) Public Chapultepec Park, Mexico City, as ornamental plants.

The collection of samples was directed to only plants with symptoms describe above (Figures 1(a), 1(b)), from June to July 2017. Eighty young agave plants (40–50 cm tall) with symptoms of yellow streaks, which formed long broad spots when they fused, and mottle or irregular whitish-yellow spots that contrast with the blue-green ash color typical of this agave species were collected from commercial plots in Tequila, Jalisco state (20°51′34″N; 103°50′5″W), the region with the highest production of tequila (DO). In addition, from September 2020 to October 2024, 25 A*. tequilana* plants used as ornamental plants were collected in Chapultepec National Park, Mexico City (CDMX) (19°24′7″N; 99°13′15″W). All plants had the same symptoms as those described above, consistent with a putative viral infection, that we call generically “Agave yellow streak,” whose causal agent has not been previously described in plants grown under natural conditions in Mexico. Finally, five asymptomatic *A. tequilana* plants grown from seeds were used as negative controls in the RT-PCR assays.

### 2.2. HTS of Viruses in Blue Agave Plants

Total RNA was obtained from 0.1 g of agave leaves exhibiting yellow streak symptoms collected in the state of Jalisco and in Chapultepec Park, CDMX with RNATrizol (Thermo Fisher Scientific, USA). We formed three mixed samples composed of tissue from five agave plants each. We sequenced three mixed samples of plants from Jalisco and three mixed samples of plants from Chapultepec Park. RNA integrity and concentration were determined by a microchip Bioanalyzer 2100 (Agilent Technologies, Santa Clara, CA), which uses the algorithm RIN (RNA integrity number), based on the degradation and concentration of the corresponding bands of ribosomal RNA 18s and 28s, which are separated by electrophoresis [13]. Libraries were constructed with the TruSeq Stranded mRNA kit (Illumina), and sequencing of RNA messengers was performed at the Unidad Universitaria de Secuenciación Masiva del ADN-UNAM (UUSMD) of the Instituto de Biotecnología (IBT) UNAM, campus Cuernavaca, Morelos, with the platform ILLUMINA Next-seq 500 platform, using paired ends, a library size of 300 pb and a reading size of 75 pb [14]. All the sequences obtained were filtered and cleaned with Trimmomatic Ver. 0.32. The quality of HTS was evaluated by Fastqc Ver. 0.12 with a loss of 1% of the total reads obtained. The reads were mapped onto a database of only viruses obtained from GenBank [15], using the software BBMap Ver. 38.26. The mapped and unmapped reads were assembled de novo using the software Trinity Ver. 2.8.4 [16]. The assembled contigs were analyzed with BLASTx to identify viral genomes [17]. Total of reads were analyzed by comparison with complete virus genomes (mapping) obtained from the GenBank (NCBI) using the software BBMap Ver. 38.26 [18]. Moreover, the reads that were not assigned to known viruses were assembled separately and analyzed by BLASTX to identify unknown viral genomes, permitting the search for genomes of other viruses [17].

### 2.3. Phylogenetic Analysis and Construction of Dendograms

All dendrograms were constructed with the software MEGA 11 [19] applying the neighbor-joining method, the GTR (general time-reversible) model, and 1500 bootstrap pseudoreplicates The analysis included the complete genomes of *Vitivirus* isolates Jalisco-1 and Jalisco-2, as well as agave *Tepovirus,* which was analyzed alongside 48 viral sequences from the Betaflexiviridae family. Additionally, the study incorporated agave potexvirus 1 isolates Jalisco and CDMX, with 54 viral sequences from the Alphaflexiviridae family. Furthermore, the *Agave badnavirus A* isolate Jal1 was included, containing 25 viral sequences from the *Badnavirus* genus. For the capsid gene dendrograms, a dendrogram (Geneious Bioinformatics software (https://www.geneious.com) with the Jukes–Cantor method) and GTR model with sequences of *Vitivirus* and *Tepovirus* obtained by RT-PCR were used, along with 19 sequences from other viruses. Finally, for the *Potexvirus* CP gene dendrogram, the sequence obtained by RT-PCR was used along with 12 sequences from other *Potexvirus* species. The accession numbers of all viral sequences downloaded from GenBank for phylogenetic purposes were also included in the dendrograms.

### 2.4. RT-PCR of the Agave Virus CP

To confirm the identity of the viruses obtained by HTS, we designed several sets of primers (Table 1) based on the analysis of the sequences of the complete genome of the identified viruses to amplify highly conserved regions of the CP of the viruses detected in the viral genome (Table 1). Total RNA obtained from 0.1 g of leaves from individual agave plants shows yellow streak symptoms with the kit RNATrizol (Thermo Fisher Scientific, USA). Ten plants from Jalisco and 25 plants from Chapultepec Park were used as template for the one-step RT-PCR assay using the SuperScript III One-Step RT-PCR System with Platinum (Invitrogen, Carlsbad, California, USA) with each oligonucleotide. The conditions of the RT-PCR were 50°C for 30 min and 94°C for 5 min of reverse transcription, denaturation at 94°C for 30 s, hybridization at 51.5°C for 1 min, and extension at 72°C for 1 min over 35 cycles and a single 10 min cycle at 72°C. The obtained RT-PCR products were analyzed by electrophoresis in 1.9% agarose gels, and molecular weight (MW) was calculated by comparison with the MW of 1 kb plus (GIBCO BRL) included in the same gel. Electrophoresis was carried out at 100 V/35 min at room temperature. The RT-PCR products obtained were sequenced by the Sanger method [20] and incorporated into the GenBank database to obtain accession numbers and later compare them with the sets of sequences obtained by HTS (NCBI/GenBank). We cloned and sequenced three of the RT-PCR products. The probable new strains of a *Vitivirus* were cloned using (NEB PCR cloning Kit, BioLabs, New England) and sequenced by the Sanger method. Their sequences were compared with those obtained by HTS and those from Sanger's CP method. Dendrograms with the cloned and noncloned sequences of the CP were then constructed.

## 3. Results and Discussion

### 3.1. HTS of Viruses in Blue Agave Plants

#### 3.1.1. Sequence Analysis of *Vitivirus*

Three samples, each made up of a mix of 5 samples of *A. tequilana* from Jalisco, and three samples, each composed of 5 samples of agaves from Chapultepec Park, were sequenced by RNA-seq. We obtained 120 million raw reads (20 million per sample) with a size of 75 pb. For the Jalisco plants, we obtained three contigs of 6919–6949 nt of a complete *Vitivirus* genome. By pairwise comparison of nucleotide sequences, two of them (KY190215; PP216715) showed similarities of 64.5% and another (OR574034) was 64.28% similar to sequences of *Agave tequilana vitivirus 1* (MW328756.1) [21] and *Agave vitivirus A* (MH898468.1), respectively. According to the species demarcation criteria for *Vitivirus* (Fam: Betaflexiviridae) of ICTV (International Committee on Taxonomy of Virus; https://ictv.global/report_9th/RNApos/Betaflexiviridae) (< 72% nucleotide identity or < 80% amino acid identity between their CP or polymerase genes), the results suggest that one of the contigs obtained from Agave tequilana, designated as Agave yellow streak virus Jalisco-1 (AYSV-Jalisco1), may represent a previously unreported strain of *Vitivirus*. This strain was found exclusively in commercially cultivated *A. tequilana* in Jalisco. Nine similar *Vitivirus* contigs were detected in symptomatic agave plants collected in Mexico City, one of which, a 6925 nt complete genome (OR574033), by pairwise comparisons of nucleotide sequences showed similarities of 79.0%–91.5% with MW328756.1, MH898468.1, and OR574034 *Vitivirus* (isolated from Jalisco plants), respectively.

**Figure 2:**
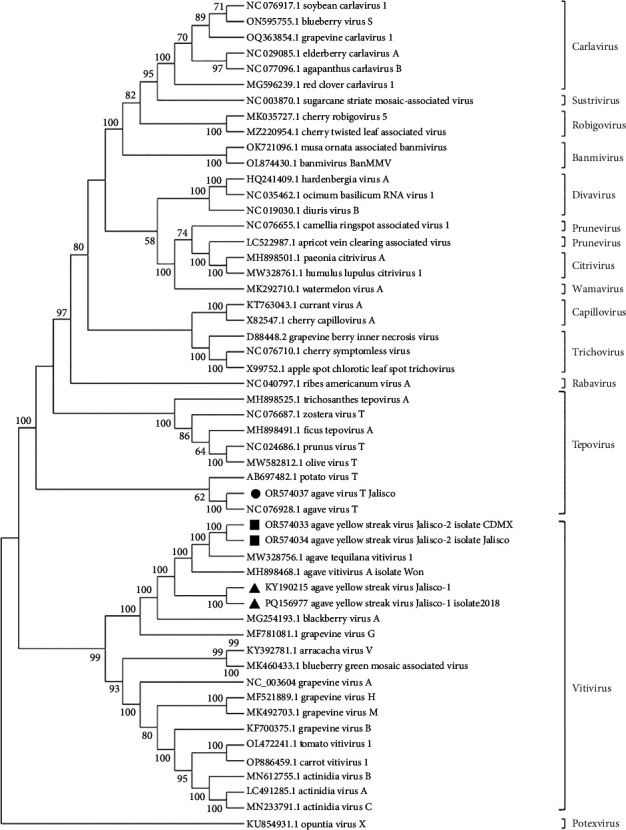
Dendrogram of the complete vitivirus and tepovirus genomes, with 1500 bootstrap pseudoreplicates. Grouping of the direct sequences of AYSV-Jalisco-1 from Mexico City and Jalisco with the other two vitiviruses previously reported and AYSV-Jalisco-2 can be seen. Also, avt from Jalisco grouped with agave virus t. potexvirus is the external group.

The phylogenetic analyses performed with the complete genome sequence of the Betaflexiviridae family and the genomes of the viruses in agaves from Jalisco (KY190215, PQ156977, OR574034) and from Mexico City (OR574033) identified the viruses as *Vitiviruses*, and *Tepovirus* was identified only in agaves from Jalisco (OR574037). The AYSV-Jalisco1 (KY190215 and PQ156977) isolated from Jalisco plants clustered on a unique clade. *Agave yellow streak virus Jalisco 2* (AYSV-Jalisco2) (OR5740343 from Jalisco and OR574034 from Mexico City) clustered with other agave *Vitiviruses*, MW328740 and MW328756.1. These results supported the proposal that *Agave yellow streak virus Jalisco 1* is a different strain of *Agave yellow streak virus Jalisco 2, Agave vitivirus 1*, and *Agave vitivirus A* and that these last three are the same virus (Figure 2).

#### 3.1.2. Sequences Analysis of *Tepovirus*

A contig that corresponds to the complete *Tepovirus* genome (OR574037) was identified in the Jalisco sample, and the pairwise comparison of nucleotide sequence showed 86% similarity to the NC076928 *Agave virus T* (AVT) [22]. Considering the species demarcation criteria for *Tepovirus* (Fam: Betaflexiviridae, ICTV) < 72% nt identity (or 80% aa identity) between their CP or polymerase genes, the *Tepovirus* obtained in agave samples from Jalisco is the same as that previously identified by Goh et al. The phylogenetic analysis of Betaflexiviridae family showed that these sequences clustered with other *Tepovirus*, forming a clade with AVT (Figure 2). These results supported the identity of a *Tepovirus* isolated from agave plants only from Jalisco is the same virus as AVT.

#### 3.1.3. Sequence Analysis of *Potexvirus*

**Figure 3:**
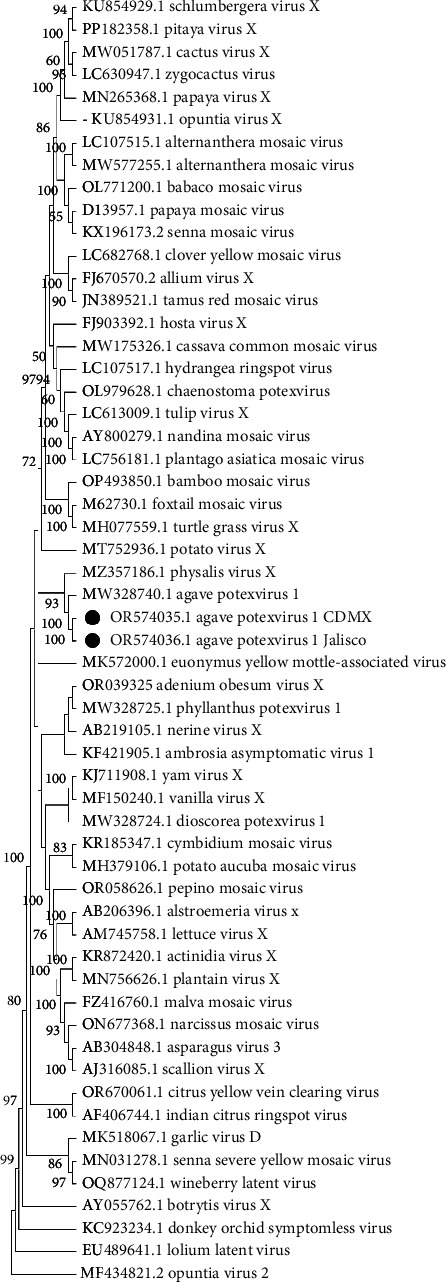
Dendrogram of the complete potexvirus genome with 1500 bootstrap pseudoreplicates. Agave potexvirus 1 from Mexico City and Jalisco is grouped with agave potexvirus 1. Tobamovirus is the external group.

We identified complete genomes of a *Potexvirus* (OR574035 and OR574036) from Mexico City and Jalisco samples, respectively. The pairwise comparisons of nucleotide sequences showed 86% similarity to the sequence MW328740.1 agave potexvirus 1 [21], and according to the species demarcation criteria (< 72% in nucleotides and < 80% in amino acids in CP and RdRp) (Fam: Alphaflexiviridae ICTV; https://ictv.global/report_9th/RNApos/Alphaflexiviridae), they are the same species. Likewise, the phylogenetic study conducted with the complete genome sequence of the viruses from the Alphaflexiviridae family, together with the complete genome sequence of the sequences obtained by HTS of agave potexvirus 1 in Mexico City, MW328740.1 (Figure 3) previously reported in agave RNA-seq data [21], confirmed the identity of agave potexvirus 1 isolated from *A. tequilana* in Mexico.

#### 3.1.4. Analysis of Partial Sequences of *Badnavirus* and Other Viruses

**Figure 4:**
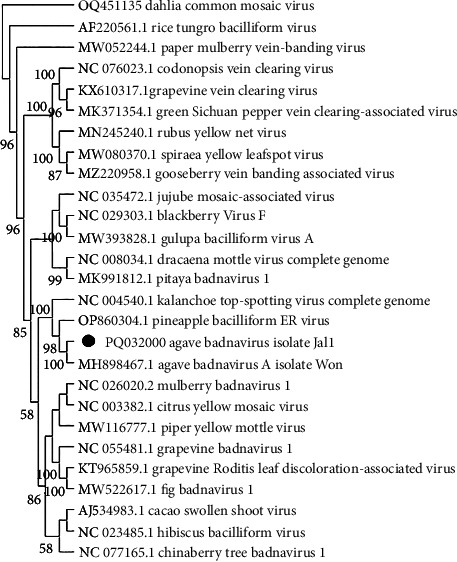
Dendrogram of the complete badnavirus genome with 1500 bootstrap pseudoreplicates. Agave badnavirus a from Jalisco is grouped with other badnavirus. Dahlia common mosaic virus is the external group.

Pairwise comparison of a sequence of the partial genome of a *Badnavirus* (OR574038.1 and PQ032000) from Jalisco samples revealed 90% similarity in nucleotides to the sequence MH898467.1 *Agave Badnavirus A* available in the GenBank. The species demarcation criterion is > 20% difference in the polymerase gene; therefore, the *Badnavirus* obtained is the same as the one previously reported. The phylogenetic analysis confirmed its identity with the genus *Badnavirus*, as it clustered with *Kalanchoe Badnavirus* and *Pineapple Badnavirus* clade (Figure 4). We identified other contigs that were related to viruses; these are presented in supporting information (Supporting information Table 1).

The viruses detected and identified in *Agave tequilana* plants grown in natural commercial conditions in Jalisco state and in agave plants used as ornamentals in Chapultepec National Park indicated the presence of a viral complex associated with symptoms of streaking and yellow mottling, in which the presence in Mexico of seemingly new, previously unreported, strains of *Vitivirus* and other viruses were outstanding. We named this complex preliminarily Agave yellow streak virus Jalisco_1, the most frequently found in the samples from Jalisco and Chapultepec Park. Moreover, we identified sequences of strains of *Potexvirus* and *Tepovirus* (Order: Tymovirales) and a *Badnavirus* (Family: Caulimoviridae. DNA Reverse Transcribing genome) not previously reported in Mexico. All these viruses should be characterized in greater depth and their distribution in Mexico and in other agave species determined. Even in Mexico, the center of origin, it is unknown whether its numerous species are infected with these or other still unknown viral strains or species.

### 3.2. RT-PCR of the Agave Virus CP

**Figure 5:**
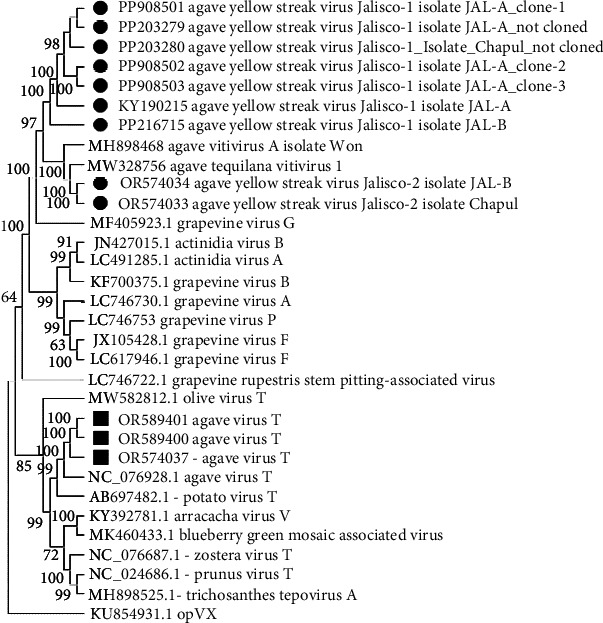
Dendrogram of the CP gene for vitivirus and tepovirus, with 1500 bootstrap pseudoreplicates. Sequences of the agave yellow streak Jalisco 1 from Mexico City and Jalisco are grouped themselves and with the others agave vitivirus. Also, AVT from Jalisco is grouped with the AVT. Elderberry carlavirus A is the external group.

**Figure 6:**
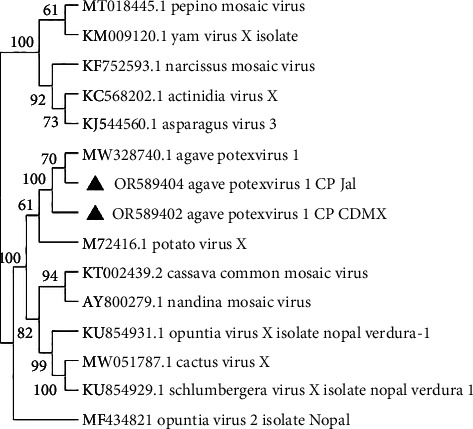
Dendrogram of the partial CP gene for potexvirus, with 1500 bootstrap pseudoreplicates. Sequences (Sanger) of agave potexvirus 1 from Mexico City and Jalisco are grouped with agave potexvirus 1. Opuntia virus 2 is the external group.

Using Sanger's method, we sequenced amplicons of the partial CP gene of *AYSV-Jalisco1* (562pb) obtained by RT-PCR from 80% of symptomatic plant samples from Jalisco (PP203279 *Agave yellow streak virus-Jalisco-1* consensus sequence) and 60% of symptomatic agave ornamental plant samples from a public garden in Mexico City (PP203280 *Agave yellow streak virus-Jalisco-1* consensus sequence), which showed 91% similarity to the genomes KY190215 *Agave yellow streak virus-Jalisco-1* isolate Jal2 and PP216715 *Agave yellow streak virus-Jalisco-1* isolate Jal1 and 68% similarity to OR574034 *Agave yellow streak virus-Jalisco-2* isolate Jal and OR574033 *Agave yellow streak virus-Jalisco-2* isolate CDMX obtained with HTS in pairwise comparison of nucleotide sequences. No amplicons were obtained from asymptomatic plants obtained from seed. Three RT-PCR products of the *Vitivirus* CP from Jalisco were cloned (NEB PCR cloning Kit, BioLabs, New England) and sequenced by the Sanger method and their sequences (PP908501, PP908502, PP908503) compared with those obtained by HTS and with those not cloned, resulting in pairwise comparison similarity of 91% nt and 95% aa to KY190215 and PP216715 and 68%–70% nt and 70%–71% aa similarity to OR574034 and OR574033. Analysis of the whole viral genome of sequences from agave showed a genomic organization that is typical of the members of the *Vitivirus* genus (Order: *Tymovirales*) [23] and described its genomic map with the position of the different open-reading frames (ORF) and their noncoding regions (UTR). According to the species demarcation criteria proposed by ICTV [15] for *Vitivirus* (less than about 72% nt identity or 80% aa identity between their CP or polymerase genes), it could be considered a strain of *Vitivirus* different from those described so far. This coincides with one of the *Vitivirus* genomes found in *Agave tequilana* from Jalisco, Mexico, that we tentatively named *Agave yellow streak virus Jalisco-1*. RT-PCR products of *Tepovirus* and *Potexvirus* were obtained in the expected size and sequenced by Sanger's method, and dendrograms were constructed. The CP nucleotide sequences obtained by HTS, cloned, and noncloned RT-PCR products were compared in a dendrogram (Geneious Bioinformatics software (https://www.geneious.com) with the Jukes–Cantor model) and grouped according to sequences of the genus *Vitivirus* available in GenBank [22] (Figure 5). For the AVT, amplicons was obtained in the Jalisco sample (OR589400 and OR589401), which showed a pairwise comparison similarity of 100% in nt and 100% in aa, respectively, with the sequence obtained by HTS (OR574037) (Figure 5). For *Agave potexvirus_1*, amplicons (OR589404) were obtained in the Jalisco samples (OR589402) and in the Chapultepec samples (OR589403), which showed a 98% nt and 100% aa pairwise comparison similarity, respectively, with the sequence obtained by HTS (OR574036) (Figure 6). However, it was not possible to obtain PCR products for sequencing a *Badnavirus* and only partial sequences of the genome of this virus were obtained by HTS, and so the complete genome of this *Badnavirus* found in the agave samples from Jalisco must be determined by methods other than HTS. In summary, mixed infections were present in 100% of the plants tested. The type of cultivation of the agave plant by vegetative propagation could be a reason for the presence of mixed infections, by cloning a plant infected with certain viruses in several plants with the same viruses. Although we have not assessed the economic impact of viral infections in *A. tequilana* plants, we have observed that those in which these viruses have been identified exhibit seasons where yellow streak symptoms are evident. However, in two to three subsequent seasons, these symptoms disappear. This could suggest that the plant's immune system is adapting to the viral infection (data not shown).

For most studies in plant virology, it is essential to be able to establish a proper diagnosis, that is, to recognize a disease and identify precisely the causal virus or viruses in multiple viral infections, which are now more commonly detected than before. To characterize the etiology of a new viral disease, it is necessary to identify the virus, reveal its presence in an infected plant, and verify that it is actually the agent responsible for the symptoms following the rules of Koch's postulate. Highly efficient, quick, and economical diagnostic techniques are also needed to study the epidemiology of a viral disease, to evaluate the efficiency of control methods or to guarantee that plants or seed are virus-free [24]. This involves performing several individual tests. Using bioassays, electron microscopy, ELISA, RT-PCR, and RT-qPCR [25] can be challenging for highly variable viruses, and so it may be difficult to design a universal assay that detects all known and unknown variants. By contrast, HTS is a comprehensive single test that can detect all viruses, including novel variants [26, 27]. To the best of our knowledge, this is the second study in which HTS has been used with *Agave* plants as a resource for virus identification. Quintanilha-Peixoto et al. [28] identified approximately 25 viral species in *A. sisalana* plants; of these species, we identified the *Closterovirus* in our samples (supporting information 1). Previously, in Mexico, an isolate of *Tuberose mild mosaic virus* (*Potyvirus*) was detected and characterized in *A. attenuata* and *A. amica*, with symptoms of green mosaic and streak, using HTS. This virus was detected only in these two species of agave, but not in *A. tequilana* [29]. The identification of novel and unique viruses, such as vitiviruses, tepoviruses, and potexviruses, in *A. tequilana* plants, but not in other agave species, suggests that human handling may be facilitating the transmission of these viruses to *A. tequilana* from other plants, where they may have subsequently adapted.

This demonstrates the potential of HTS for identifying new viruses and highlights its ability to compare viruses in both wild and cultivated environments.

The greatest advantage of HTS over other diagnostic approaches is that it gives a complete overview of the viral phytosanitary status of a plant. In theory, HTS can detect all viruses in a single assay, and performance is limited only by the completeness of the reference database (s) against which the sequences are compared. Sequence information obtained can also be used to provide insight into virus population structure, ecology, or evolution or to differentiate virus variants that may contribute differently toward disease etiology. HTS has the potential to reduce the time from virus discovery to development of targeted detection assays such as PCR or LAMP and to contribute to the improvement of existing assays, by elucidating sequence variation within virus populations. Another advantage of HTS is that sequence data can be analyzed by multiple end-users or may be re-analyzed as databases are expanded [10].

In this contribution, using HTS, we identified four viruses and two variants not previously identified in Mexico in *A. tequilana* with symptoms of striping and yellow mottling. The biological tests, ELISA, PCR, or RT-PCR for the known viruses, could not identify the viruses possibly related to the yellow stripe and mosaic symptoms. However, the flexible rod particles observed by TEM would confirm the presence of the viral genera: *Vitivirus, Tepovirus*, and *Potexvirus*. Considering the sequences obtained by HTS, specific oligonucleotides were designed for the CP regions for the identified viruses and sequenced, and the obtained products were cloned, thus confirming the identity of the viruses associated with *A. tequilana* yellow stripe. Mexico is the center of origin and primary diversification of 200 species of *Agave*, and although in some species symptoms like those caused by viruses have been observed, it is unknown at this time whether these viruses or other unknown viruses are affecting other species. The strategy of using HTS to determine the virome of Agave species would allow us to know the extent of their distribution, the diversity of possible new species or variants of viruses in this genus of plants, the damage they cause, and perhaps their possible relationship with other species of cultivated or even wild plants.

## 4. Conclusions

The symptoms of *Agave yellow stripe virus* are related to infections in a mixture of different species of RNA viruses and a DNA virus from a symptomatic leaf identified by HTS analysis and by sequencing cloned and noncloned RT-PCR products using CP-specific oligonucleotides derived from HTS sequences. The results confirmed the identity of a different strain of a *Vitivirus*, preliminarily named *AYSV-Jalisco-1*. HTS proved to be a very efficient technique in detecting unknown viruses in *A. tequilana* and in confirming the presence of others already partially described. This means that this technique could be used to detect viruses in other agave species, which are numerous and of great importance to Mexico as the center of origin or primary diversification. These viruses could be a threat to these plants and potentially to other susceptible crops. To our knowledge, this is the first report of a mixed viral infection associated with yellow mottle streak symptoms in *Agave tequilana* in commercial plantations and nurseries in Mexico.

## Figures and Tables

**Table 1:** Specific oligonucleotides to amplify a conserved region of the capsid protein (CP) of the viruses detected by HTS.

Virus	Primer	Sequence 5′ to 3′	Size	Region (nt)	Tm
Vitivirus	Agavteq-viti-5, 529F	ATACTGACTCTGCTGAGCGC	500 pb	CP, 5529–6095	55°C
	Agavteq-viti 6, 095R	CTCCACCGAGTTTTCCCCAA			

Potexvirus	Potex agavteq 5, 670F	TGACAATGCCAAAGCCGTTG	500 pb	CP, 5670–6410	55°C
	Potex agavteq 6, 410R	TTGCCCTGTGTAATCCGGAC			

Tepovirus	Tepovirus agavteq 5, 280F	ACGGCCACAATGTACTCAGG	500 pb	CP, 5280–5888	57°C
	Tepovirus agavteq 5, 888R	TCTCTCCCCGAATCTTGCG			

Badnavirus	Agave_badna-1, 785F	AACATCGGCCGAACCATCAT	639 pb	ORF3, 1785–2418	55°C
	Agave_badna-2, 418R	TAGAGTGCAGGTATTCGGCG			

## Data Availability

The accession number of the complete genomes sequences for all viruses reported (KY190215, OR574037, OR574036, PQ115117, PQ156977, OR574038, PQ032000, OR574034, PP216715, PQ184855, PQ184856, OR574033, PQ184857, PQ184858, OR574035, PQ115119, and PQ184859) is available in the National Center for Biotechnological Information (NCBI). The accession numbers of partial sequences of the *Vitivirus* (PP203279, PP203280, PP908501; PP908502; PP908503), *Potexvirus* (OR589402, OR589403, and OR589404), and *Tepovirus* (OR589401) are available in the National Center for Biotechnological Information (NCBI). Any other relevant data may be available upon request from the first author. The accession number for the raw sequencing data: PRJNA1245605.

## Supporting Information

Table 1. Corresponds to the table where all the viral contigs identified in the samples during the bioinformatic analysis and that were registered in GenBank are included.
