# Supplementary material for: High-Throughput Sequencing Detects a Viral Complex in Agave Tequilana Plants
Source: Adv Virol. 2025 May 4;2025:6434701. doi: 10.1155/av/6434701 (PMC12066183; doi:10.1155/av/6434701)
Supplement: Supporting Information — Additional supporting information can be found online in the Supporting Information section. [file 6434701.f1.docx]

**Supplementary material Table 1**. Viral contigs assembled de novo and identified per sample.

| **agave sample** | **Location** | **Year** | **virus family/genus** | **virus name** | **lenght (nt)** | **Accesion number** | **ORF (nt position)** | **sequence_compared** | **similarity to** |
| --- | --- | --- | --- | --- | --- | --- | --- | --- | --- |
| L8P1_Agave_tequilana_M1 | Jalisco | 2016 | vitivirus  Betaflexiviridae | Agave yellow streak virus Jalisco 1 | 6958 | KY190215 | RdRp:53..4672,Hypothetical protein:4673..5128,MP:5129..5932,CP:5862..6461,NbP:6492..6872 | MW328756 | 64.4 % |
|  |  |  | tepovirus  Betaflexiviridae | Agave virus T | 6046 | OR574037.1 | RdRp:54..4505,MP:4414..5481,CP:5261..5923 | [MW323519](https://www.ncbi.nlm.nih.gov/nuccore/MW323519) | 86.76% |
|  |  |  | potexvirus/  Alphhaflexiviridae | Agave potexvirus 1 | 6559 | OR574036.1 | RdRp:105..4439,TGB1:4473..5150,TGB2:5131..5520,TGB3:5327..5635,CP:5602..6444 | MW328740.1 | 81.87% |
|  |  |  | closterovirus/  Closteroviridae | Sisal_associated closterovirus | 2345 | PQ115117 | CP minor: 83..1507, CP major:1564..2088 | MZ599629.1 | 97.67% |
| L10P119_Agave_tequilana_M2 | Jalisco | 2020 | vitivirus/  Betaflexiviridae | Agave yellow streak virus Jalisco 2 | 6931 | PQ156977 | RdRp:48..4646,Hypothetical protein:4646..5101,MP:5115..5924,CP:5848..6447,NbP:6472..6834 | [MH898468.1](https://www.ncbi.nlm.nih.gov/nucleotide/MH898468.1?report=genbank&log$=nucltop&blast_rank=1&RID=AJR4UW3K016) | 97.2 % |
|  |  |  | badnavirus/  Caulimoviridae | Agave badnavirus A | 3824 | OR574038.1 | ORF3:4446..8276 | MH898467.1 | 97.13% |
|  |  |  | badnavirus/  Caulimoviridae | Agave badnavirus A | 6693 | PQ032000 | ORF1:734..1264,ORF2:1261..1647,ORF3:1644..6693 | MH898467.1 | 73.58% |
| L12P181M3_Agave_tequilana | Jalisco | 2023 | vitivirus/  Betaflexiviridae | Agave yellow streak virus Jalisco 2 | 6936 | OR574034.1 | RdRp:53..4657,Hypothetical protein:4657..5112,MP:5126..5935,CP:5859..6458,NbP:6476..6862 | MW328756.1 | 88.30% |
| L12P148M4_Agave_tequilana | Jalisco | 2023 | vitivirus/  Betaflexiviridae | Agave yellow streak virus Jalisco 1 | 6969 | PP216715 | RdRp:42..4667,Hypothetical protein:4668..5123,MP:5124..5927,CP:5857..6456,NbP:6487..6867 | KY190215.1 | 92.50% |
|  |  |  | tepovirus/  Betaflexiviridae | Agave virus T | 6418 | PQ184855 | RdRp:50..4867,MP:4746..5843,CP:5590..6285 | MW328727.1 | 86.76% |
|  |  |  | potexvirus/  Alphaflexiviridae | Agave potexvirus 1 | 6598 | PQ184856 | RdRp:130..4464,TGB1:4498..5175,TGB2:5156..5545,TGB3:5352..5660,CP:5627..6457 | MW328740.1 | 81.92% |
| L10P164_Agave_tequilana_M17 | Chapultepec,CDMX | 2021 | vitivirus/  Betaflexiviridae | Agave yellow streak virus Jalisco 2 | 6934 | OR574033.1 | RdRp:67..4665,Hypothetical protein:4665..5120,MP:5134..5943,CP:5867..6466,NbP:6484..6870 | MW328756.1 | 88.35% |
|  |  |  | potexvirus/  Alphaflexiviridae | Agave potexvirus 1 | 6571 | PQ184857 | RdRp:93..4436,TGB1:4470..5147,TGB2:5128..5517,TGB3:5324..5632,CP:5599..6429 | MW328740.1 | 83% |
| L12P148M3_Agave_tequilana | Chapultepec,CDMX | 2023 | vitivirus/  Betaflexiviridae | Agave yellow streak virus Jalisco 1 | 6952 | PQ184858 | RdRp:42..4667,Hypothetical protein:4668..5123,MP:5124..5927,CP:5857..6456,NbP:6487..6867 | KY190215.1 | 89.07% |
|  |  |  | potexvirus/  Alphaflexiviridae | Agave potexvirus 1 | 6598 | OR574035.1 | RdRp:130..4464,TGB1:4498..5175,TGB2:5156..5545,TGB3:5352..5660,CP:5627..6457 | MW328740.1 | 81.92% |
|  |  |  | potyvirus/  Potyviridae | Agave tequilana agavirus | 2055 | PQ115119 | Polyprotein:7197..9250 | MZ682615.1 | 85.13% |
|  |  |  | Alphanucleorhabdovirus/  Rhabdoviridae | Agave tequilana virus 1 | 1024 | PQ184859 | L:11746..12764 | BK014297.1 | 99.61% |
